# Minutes of the Cincinnati Local Dental Association

**Published:** 1861-02

**Authors:** T. F. Davenport


### Proceedings of Societies.

### MINUTES OF THE CINCINNATI LOCAL DENTAL ASSOCIATION.

CINCINNATI, Jan. 8, 1861.

Local Dental Association met pursuant to adjournment, at the Dental College, Vice-President in the chair.

Members present: Drs. H. R. Smith, Taft, Taylor, Richardson, Wardle, Wells, Kendrick, Wheeler, Cameron, Bonsall, James, Irwin and Davenport.

Minutes of previous meeting read and approved.

On motion of Dr. Richardson, the rules were suspended, for the purpose of hearing Dr. Taylor's paper on the Life and Character of the late Prof. C. A. Harris.

A vote of thanks was tendered to Dr. Taylor, and a copy of his paper requested for publication.

The committee on a Code of Ethics made a report, which was accepted, and discussed by most of the members present, and finally referred back to the committee, with instructions to re-report it (with alterations) at a called meeting, January 15, 1861.

The proposition in regard to a change in the name of the Association was then taken up, and discussed at length, by the members. Upon the final vote, the proposition not receiving the support of two-thirds of the active members, was lost.

Adjourned to meet on Tuesday evening, Jan. 15, 1861.

T. F. DAVENPORT, *Sec'y.*

